# Ecological niche modeling of genetic lineages of the great gerbil, *Rhombomys opimus* (Rodentia: Gerbillinae)

**DOI:** 10.1371/journal.pone.0257063

**Published:** 2021-09-02

**Authors:** Kordiyeh Hamidi, Saeed Mohammadi, Taghi Ghassemi-Khademi

**Affiliations:** 1 Department of Biology, Faculty of Science, Ferdowsi University of Mashhad, Mashhad, Iran; 2 Department of Environmental Sciences, Faculty of Natural Resources, University of Zabol, Zabol, Iran; 3 Department of Biology, Faculty of Sciences, University of Shiraz, Shiraz, Iran; Qinghai University, CHINA

## Abstract

Great gerbil (*Rhombomys opimus* Lichtenstein, 1823) is distributed in Central Asia and some parts of the Middle East. It is widely found in central and northeast parts of Iran with two distinct genetic lineages: *R*. *o*. *sodalis* in the northern slopes of the Elburz Mountains and *R*. *o*. *sargadensis* in the southern slopes. This large rodent acts as the main host of natural focal diseases. No study has surveyed the ecological niche of the lineages and how their distribution might be influenced by different climatic variables. To examine the distribution patterns of this murid rodent, we aimed to determine the habitat preferences and effects of environmental variables on the ecological niche. Using a species distribution approach for modeling of regional niche specialization, suitable habitats predicted for *R*. *o*. *sodalis* were mainly located in Golestan province in northern Iran, along the northern slope of Elburz, while *R*. *o*. *sargadensis*, showed great potential distribution along the southern slope of Elburz and around the Kavir Desert and the Lut Desert. Despite the widest potential distribution of *R*. *o*. *sargadensis* from northeast to northwest and through Central Iran, the geographic range of *R*. *o*. *sodalis* was smaller and mostly confined to Golestan province. The results support the presence of the two genetic lineages of *Rhombomys* in Iran and confirm that there is no significant niche overlap between the two subspecies. Furthermore, it provided several perspectives for future taxonomic studies and prevention hygiene programs for public health.

## Introduction

Great gerbil (*Rhombomys opimus* Lichtenstein, 1823), which is generally considered to be a monotypic species based on coloration and size [1], is known to be distributed throughout Central Asia and some parts of the Middle East, including Kazakhstan, Uzbekistan, Kyrgyzstan, Tajikistan, Turkmenistan, North China, South Mongolia, Iran, northern Afghanistan, and southwest Pakistan [2, 3]. Generally, this species is abundant in harsh climatic conditions with hot and dry summers and cold winters characterized by low average annual precipitation and relative humidity [4, 5]. *Rhombomys opimus* has a social structure, and individuals often burrow close to one another; the family group (adult male, several adult females and young individuals of several generations) lives in one complex burrow system [3, 6].

This large gerbil has a wide distribution range in central and northeastern Iran and occupies sandy or clay deserts, usually in foothills and mountainous areas with scattered shrubby vegetation, especially *Haloxylon ammodendron* (Amaranthaceae), succulent plants such as *Salsola* spp., *Climacoptera* spp., and *Suaeda* spp. (Amaranthaceae) [3, 7]. The patchy distribution of saline ecological biotopes in the north, northwest and center of the country leads to isolated populations of the great gerbil, and high molecular variability within Iranian populations has been reported [1, 8]. Ellerman [9] listed seven nominal subspecies for this species, whereas the real number of described forms is 12 [2]. An accurate number of subspecies critically need to be estimated in a future taxonomic revision, but provisionally can be accepted as six: *R*. *o*. *opimus* (Lichtenstein, 1823) (Uzbekistan, western, central and southern Kazakhstan), *R*. *o*. *giganteus* (Büchner, 1889) (China: northern Xinjiang, southeastern Kazakhstan, southwestern Mongolia), *R*. *o*. *nigrescens* (Satunin, 1903) (Mongolia, China: Gansu, Inner Mongolia, southeastern Xinjiang), *R*. *o*. *fumicolor* Heptner, 1933 (Fergana Valley), *R*. *o*. *sodalis* Goodwin, 1939 (northeastern Iran, Turkmenistan, northern Afghanistan), and *R*. *o*. *sargadensis* Heptner, 1939 (central and southeastern Iran, southern Afghanistan, Pakistan) [2].

**Fig 1:**
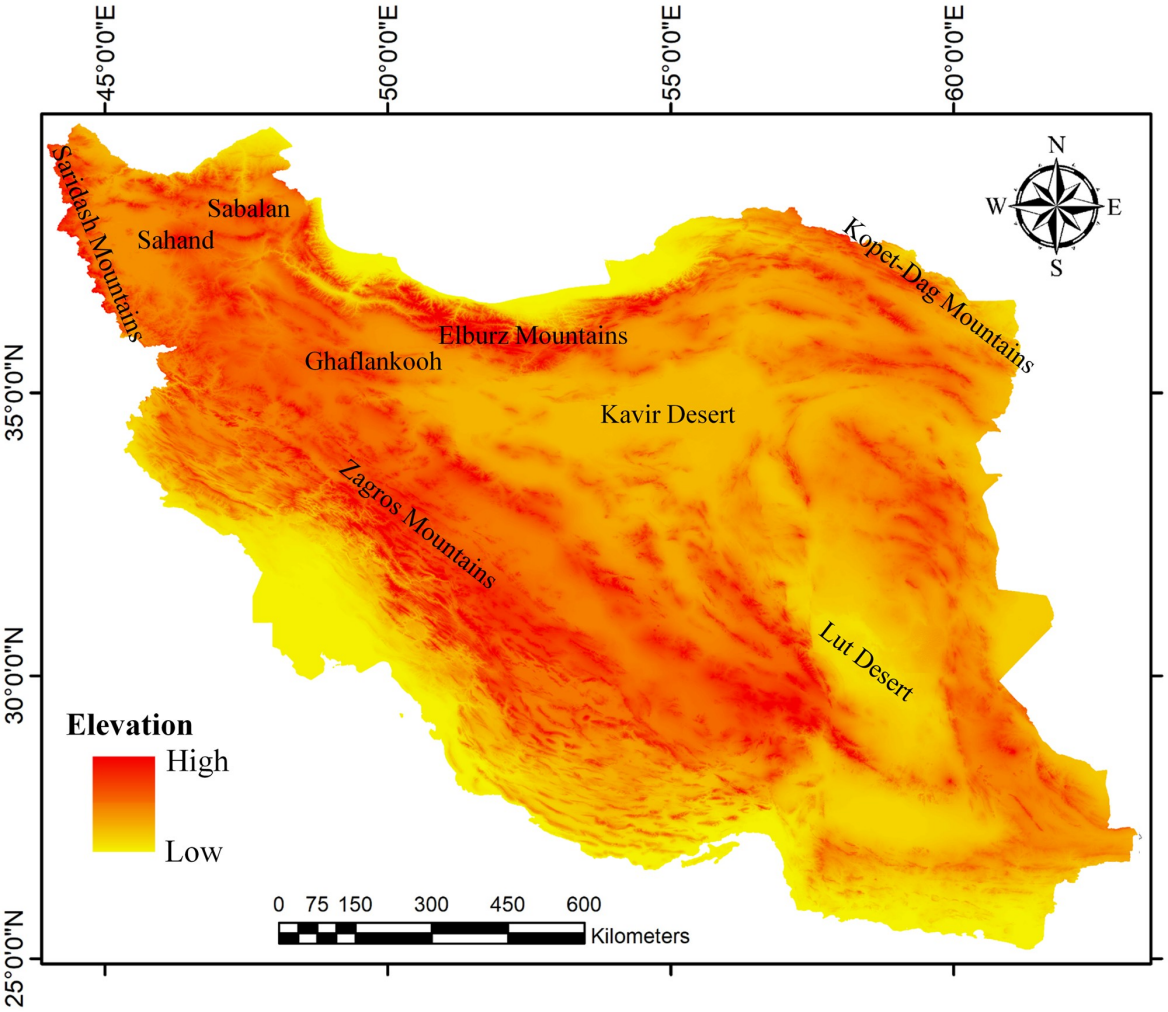
Tepographic map of Iran showing the main Iranian regions as suitable inhabitation areas and corridors for the great gerbil.

In Iran, *Rhombomys opimus sodalis* is only found at elevations of approximately 600–1000 m in Golestan province on the northern slope of the Elburz Mountains in addition to small patches in North Khorasan (Bojnourd) and Razavi Khorasan (Sarakhs and Dargaz) provinces in northeast of Iran, whereas *R*. *o*. *sargadensis*, which is considered the more widely distributed subspecies, could be found at higher elevations than *R*. *o*. *sodalis* on the southern slope of the Elburz Mountains and around the Kavir Desert in Central Iran [1, 8, 10, 11] (Fig 1).

These two subspecies are also morphologically distinct from each other with known differences in coloration and size [1]. Due to their sympatric presence in Iran, based on the report of sympatric haplotypes of *R*. *o*. *sodalis* in a population of *R*. *o*. *sargadensis* in the Shahrood district (Semnan province), near Kavir Desert [8], it has been suggested that migration of *R*. *o*. *sodalis* from Golestan province to the territories of *R*. *o*. *sargadensis* in Semnan province is possible. However, Oshaghi et al. [8] and Bakhshi et al. [10] attributed these “sympatric haplotypes” to either common ancestry or migration. Nevertheless, mtDNA markers are more likely to reflect shared ancestral populations rather than recent migration. The reciprocal crosses between individuals of the two subspecies attempted by Oshaghi et al. [8] show that there are no pre or post-zygotic barriers between *R*. *o*. *sodalis* males and *R*. *o*. *sargadensis* females. However, the reciprocal cross of *R*. *o*. *sodalis* females with *R*. *o*. *sargadensis* males resulted in the death of the paired individuals, suggesting the possibility of some prezygotic isolation mechanism. Bakhshi et al. [10] obtained offspring from this reciprocal cross, although he provides no details of the number of crosses attempted or the number of successful crosses. Moreover, the Elburz Mountain Chain in northern Iran acts as a natural barrier between populations of *R*. *o*. *sodalis* and *R*. *o*. *sargadensis* [10], and consequently, future speciation is predicted due to the large intraspecific variation among populations distributed in different localities across Iran [8].

Great gerbils can damage crops and irrigation canals, destroy the vegetation and cause the die-off of plants over their colonies by biting the main roots. Hence, they are in direct competition with livestock and have been recognized as carriers of several zoonotic diseases (e.g., plague, leishmaniasis, leptospirosis, and chronic respiratory disease) [12–14]. The presence of flea (Siphonaptera) assemblages and sandflies (Diptera) (zoonotic cutaneous leishmaniasis transmitters) in great gerbil burrow systems increases their ability to sustain the pathogen and the probability of infectious emergence of rodent-borne diseases [15–20].

With respect to the lack of information on current habitat delimitation and the potential distribution pattern of great gerbil genetic lineages (or subspecies) in Iran, we seek to elucidate their environmental niches through species distribution models. Accordingly, this study aimed to examine (i) whether the genetic lineages of *R*. *opimus* have different habitat suitability and niche specialization throughout the Iranian Plateau and/or in the contact zone, where they are found in sympatry, and also (ii) to understand the role of environmental variables on their regional spatial distributions.

## Materials and methods

### Environmental and species data

To use a machine learning model, 19 bioclimatic variables were selected from the WorldClim database (www.worldclim.org) at a spatial resolution of 1 km^2^ and then processed to model the target genetic lineage distribution. Since multicollinearity among bioclimatic variables may result in misunderstanding the contribution ratio of the most important variables to the model [21], highly correlated variables (Pearson correlation coefficient: *r*≥ 0.75) were ignored for final analysis. The final set contained 13 climatic variables, as shown in Table 1.

**Table 1:** Bioclimatic variables included in the MAXENT distribution models and relevant contributed layers for the great gerbil (*Rhombomys opimus*) subspecies.

Variables	Contribution values (%)	
	*R*. *o*. *sodalis*	*R*. *o*. *sargadensis*
Bio1 (annual mean temperature)	5.77	7.05
Bio2 (mean diurnal range)	7.95	-
Bio3 (isothermality)	8.20	-
Bio6 (mean temperature of coldest month)	-	8.19
Bio8 (mean temperature of wettest quarter)	-	6.30
Bio9 (mean temperature of driest quarter)	6.20	7.10
Bio10 (mean temperature of warmest quarter)	6.88	-
Bio12 (annual precipitation)	8.90	-
Bio13 (precipitation of wettest month)	-	14.90
Bio14 (precipitation of driest month)	10.45	-
Bio15 (precipitation seasonality)	9.20	-
Bio16 (precipitation of wettest quarter)	-	14.02
Bio19 (precipitation of coldest quarter)	-	29.08

**Fig 2:**
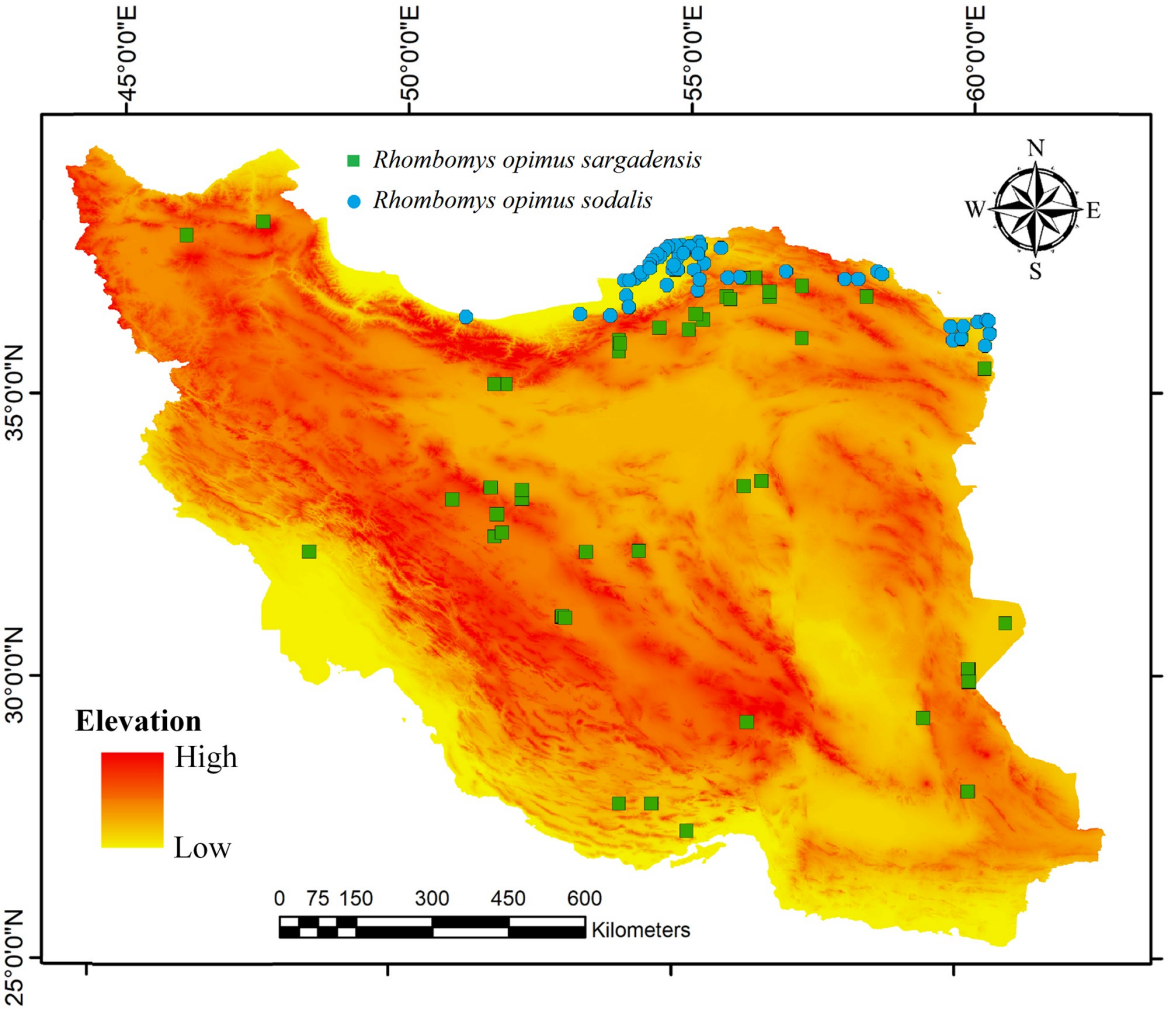
Point localities map of *Rhombomys opimus*. Known localities for *R*. *o*. *sargadensis* and *R*. *o*. *sodalis* are shown as green squares and blue circles, respectively.

Distribution data were gathered from the Global Biodiversity Information Facility (GBIF database), VertNet as a publicly accessible database of vertebrate biodiversity data from natural history collections around the world, and published papers and books [e.g., 3, 22, 23]. A total of 102 distribution records within the country were obtained (*R*. *o*. *sodalis*: n = 56 and *R*. *o*. *sargadensis*: n = 46) (Fig 2). The points were screened in ArcGIS 10 (ESRI, Redland, USA) with nearest neighbour analysis to assess spatial autocorrelation filtered using SDMTools [24, 25]. This analysis discovered a low clustering among presence records. An Excel file including point localities for *R*. *opimus* subspecies is available at S1 Appendix. No ethical approval is required for this study because techniques performed here do not involve animals.

### Modeling potential occurrence

To model the current geographic distribution range of great gerbil subspecies, the maximum entropy modeling algorithm (MAXENT v. 3.3.3. program, www.cs.princeton.edu) was used [26]. MAXENT has been found to perform better than many other modeling methods for occurrence data to predict a species’ distribution [27–29]. MAXENT is proficient of calculating species distribution using presence-only records; it can consider both continuous and discrete variables in the model to identify the important environmental variables affecting species distribution [27, 30].

MAXENT was applied, and 70% of the occurrence records were used as training data and the remaining 30% to test it (as test data). To determine the model performance, the calculated value of the area under the curve (AUC) of the receiver operating characteristic (ROC) curve on the training and testing data was considered. The AUC evaluator indicates the power of the model in distinguishing presence from absence records. A calculated value close to 1 indicates the high predictive ability of the model, while a value of 0.5 suggests that the model lacks sufficient power to predict the species distribution range [25, 31–34]. Spatial prediction maps of habitat suitability for any given location, as the model output, range from 0 (very low) to 1 (very high) relative habitat suitability of species presence [26, 28]. Jackknife analyses used to estimate the importance of each of the variables that reduce the model reliability when omitted. Moreover, ENMTools [35] was used to test the percentage of niche overlap between the two predicted models using Schoener’s *D* [36] and Hellinger’s based *I* [37] indices.

## Results

**Fig 3:**
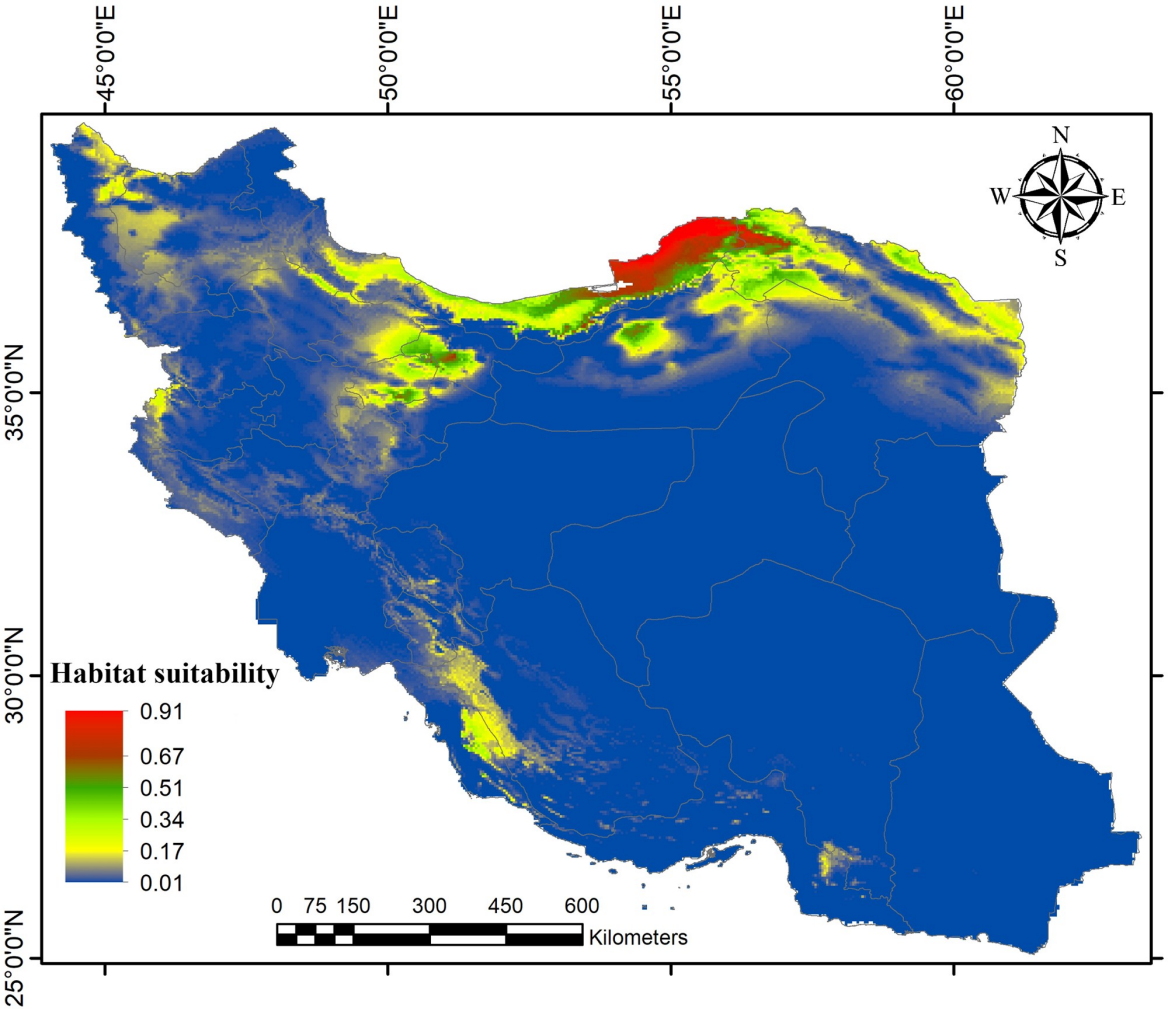
Predictive distribution map of *Rhombomys opimus sodalis* in MAXENT models.

**Fig 4:**
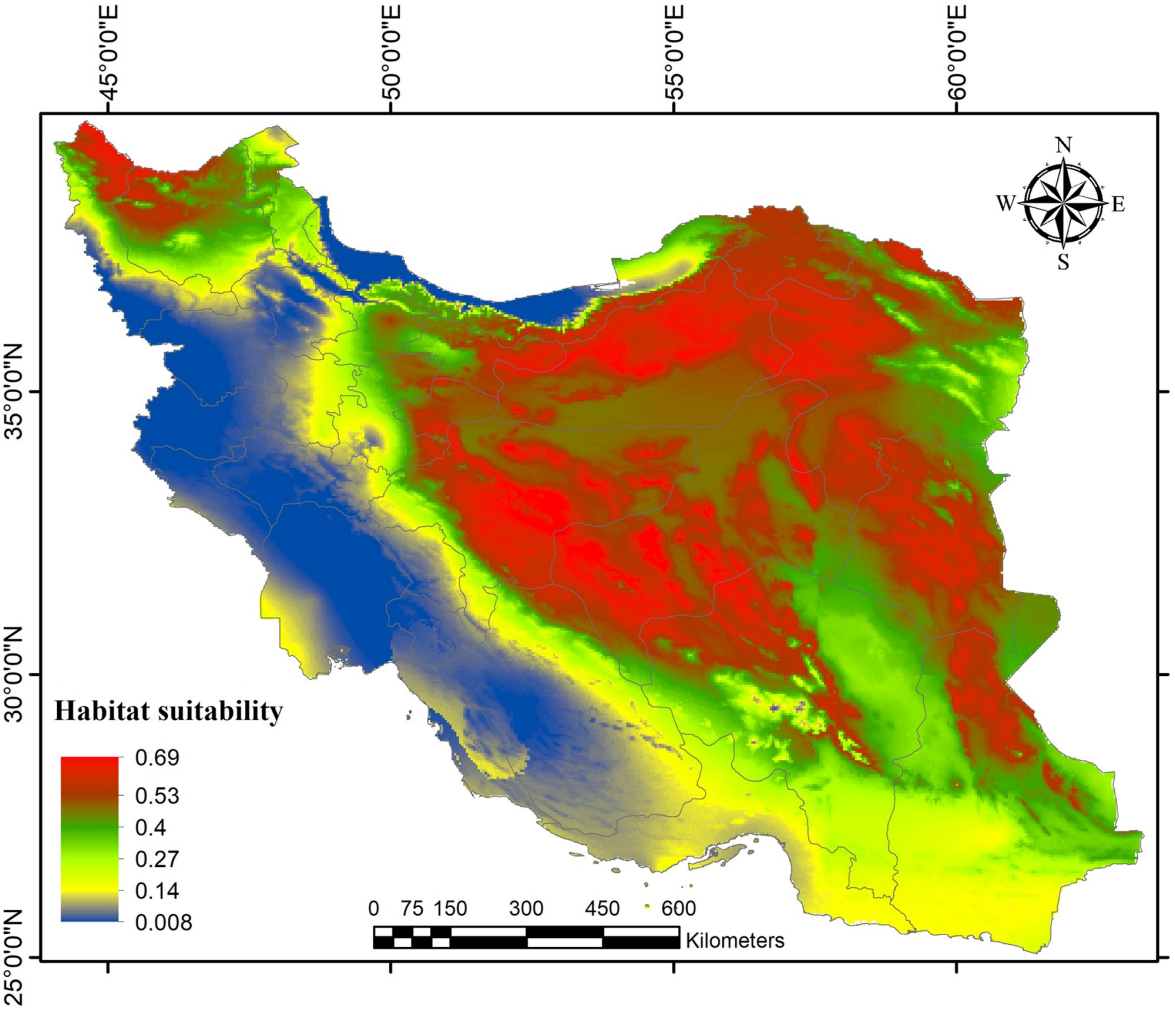
Predictive distribution map of *Rhombomys opimus sargadensis* in MAXENT models.

According to the obtained AUC values, the model predictive accuracy of both subspecies indicated high performance (AUC = 0.77 for *R*. *o*. *sargadensis*; AUC = 0.92 for *R*. *o*. *sodalis*), so the MAXENT approach seems to perform well for modeling ecological niche segregation. Based on our models, the most current suitable habitats of *R*. *o*. *sodalis* were mainly projected in Golestan province, along the northern slope of the Elburz Mountains, and penetrated further into North Khorasan provinces as well as small patches in the western parts of Iran (Fig 3). Under our projection, the other subspecies, *R*. *o*. *sargadensis*, showed great potential general distribution in most parts of central, eastern and northern Iran, along the southern slope of the Elburz Mountains and around Kavir and Lut Deserts in Central Iran, as well as northwesternmost parts of Iran (Fig 4). Species distribution models obtained by MAXENT showed that precipitation of the driest month (Bio14) and precipitation seasonality (Bio15) are the most important predictor variable determining the current distribution of *R*. *o*. *sodalis*, while the most important variables for *R*. *o*. *sargadensis* are precipitation of the coldest quarter (Bio19) and precipitation of the wettest month (Bio13) (Table 1). The results also showed that the annual mean tempreture (Bio1) and mean temperature of the driest quarter (Bio9) are the most important variables in explaining the distribution of both currently assumed mitochondrial lineages.

In addition, most suitable habitats of great gerbils of the genetic lineage *R*. *o*. *sargadensis* were situated in northeastern, northwestern and Central Iran, while the geographic range of *R*. *o*. *sodalis* was rather smaller and confined mostly to Golestan province, northern Iran. Moreover, the results of ecological niche modeling revealed that there is no significant niche overlap between the two subspecies (Hellinger’s based *I* = 0.50 and Schoener’s *D* = 0.26 for *R*. *o*. *sargadensis/ R*. *o*. *sodalis*).

## Discussion

As a desert-adapted rodent, the distribution of great gerbil is significantly associated with temperature, precipitation, terrain, vegetation and other ecological environmental factors [5]. The results showed that temperature shapes the ecological niche of both *R*. *o*. *sargadensis* and *R*. *o*. *sodalis*, followed by the annual precipitation amount. However, Gholamrezaei et al. [38] indicated slope as the main variable affecting the distribution pattern of *R*. *opimus*. Furthermore, Gao et al. [5] showed that *R*. *opimus* is distributed in the area of elevation between 200 and 600 m with a slope of 0–3 degrees, an average annual temperature from 6 to 10 °C and an annual precipitation of 120–200 mm in Xinjiang, northwest China.

According to the models obtained by MAXENT, the potential distribution of *R*. *o*. *sargadensis* was strongly constrained in an isolated habitat patch in the marginal part of the species distribution range; a patch of habitat in the extreme northwest of Iran might contain genetically isolated populations (Fig 4).

With regard to different karyological statuses that have been reported for *R*. *o*. *sodalis* from Gonbad and Bandar Torkaman, and Gorgan, considering that all studied regions are located in Golestan province and in the range of *R*. *o*. *sodalis* [1, 39], we expected differentiation of the ecological niche of the species. However, previous chromosomal analysis has indicated that both genetic lineages of *R*. *opimus* in Iran have 2n = 40 chromosomes [40, 41].

Haplotypes belonging to two different mtDNA lineages are found together in some populations of northern Iran, which makes it possible to hypothesize that there is hybridization of these two genetic lineages (but it can be proven only by analysis of nuclear genes(. While Oshaghi et al. [8] made a prediction regarding future speciation, it is dependent on retaining geographic or ecological barriers between these two subspecies that have not been identified.

Some hybrid zones at a regional scale were observed in which the two genetic lineages may be in contact (Kopet-Dag Mountains in the northeast and Ghaflankooh Mountains in northwest Iran) (Fig 1). These indicated a potential niche overlap in the distribution range of both. Subsequently, the hypothesis of future speciation within the species based on geographic variation of haplotypes among localities [8] need to be assessed.

Habitat suitability for hosts and/or vectors can have effects on the spreading of pathogens and may influence both their abundance and movements [42, 43]. Suitable areas that may lead to a higher contact rate of hosts and vectors can act as corridors for the transmission of pathogens through a larger landscape, and unsuitable habitats may act as barriers because they prevent the transmission of pathogens by hosts or vectors [33, 44]. For each of the host or vector species, these corridors and barriers can show specification, depending on their movement abilities. In the present study, these are areas along the northern and southern slopes of the Elburz Mountains and the eastern parts of the Zagros Mountains around the Kavir Desert, which have been inferred as suitable areas for inhabitation of the great gerbil. The Sabalan, Sahand, and Ghaflankooh Mountains in northwestern Iran and the Saridash Mountains on the border of Iran-Turkey are other possible functional corridors for the distribution of this species. The southern shores of the Caspian Sea, throughout Golestan province and northwest of North Khorasan province, which are covered with dense forest and trees, with developing cultivation and agriculture activities that can provide food and shelter for rodent populations, may be considered as structural corridors (See Fig 1).

Harsh climatic conditions (arid or semi-arid climates) in some parts of the Kavir Desert act as another structural barrier for great gerbil to expand its distribution throughout Iran. For the great gerbil, the Elburz Mountains in the north and the Zagros Mountains in the west of the country play major barriers against dispersal.

Lizhi et al. [45] showed that the distribution of Chinese populations of great gerbil has been altered due to human activities. A study in the southern Kyzylkum Desert (western Uzbekistan) demonstrated that the population density and the size and structure of social groups varied yearly in response to changing conditions of precipitation and temperature; years with considerable rain and snowfall during winter seem to produce enough and available succulent vegetation cover to facilitate subsequent breeding and expanding family groups [46]. The great gerbil limits reproduction to periods of rainfall and the subsequent growth of green vegetation [47–50]. Thus, breeding and survival in this species seem to greatly depend on environmental conditions rather than any other factors, such as social organization [46]. Several studies reported fluctuations in rodents and their associated parasite populations with changes in climate, habitat structure and feeding resources. For example, Ari et al. [50] noted that fluctuation in the population of fleas harbored by great gerbils can occur due to rainfall, relative humidity, and temperature; in warm moist weather, rodent hosts are more available for the growth of bacteria *Yersinia pestis*, and hence, the transmission rates of plague infection may increase.

Great gerbil is considered as the most important reservoir host of *Leishmania major* in Iran, which is transmitted by sand flies of the genus *Phlebotomus* [16, 17, 19]. Zoonotic coutaneous leishmaniasis (ZCL) due to *L*. *major* is known as one of the zoonoses increasing in Iran [17]. As an example, Rassi et al. [51] stated that the rate of infection of great gerbils to this parasite is high and may reach to 92.5% at endemic areas of ZCL in Kalaleh, Golestan province, north of Iran. In another study [16], seasonal variations of natural infection with *Leishmania* in population of great gerbils in Badrood district, Esfahan province, central Iran were surveyed. The lowest and highest infection rates were observed in summer and fall, respectively. Gerbils were found to be infected with three species of *L*. *major*, *L*. *turanica* and *L*. *gerbilli*, which transmit in the population of *R*. *opimus* in central part of Iran. *Leishmania major* infection is generally accompanied by *L*. *turanica* in infected great gerbils, showing the highest rate in fall.

The distribution of *L*. *turanica* in rodents showed coincidence with the distribution patterns of sandflies [52]. Deep burrows of great gerbils, which may extend to three meters in depth depending on the stability of the soil [53], will have more stable temperatures inside and hence are likely to increase the prevalence of ectoparasites such as fleas, which are less prone to survive low humidity and extreme temperatures [54]. This rodent species plays a role as a reservoir host for the fleas of *Xenopsylla* and *Nosopsyllus* genera [20, 55], tick *Hyaloma* [4, 56], and the mite *Ornithonyssus bacoti* [55, 57–59]. Moreover, the oxyurid *Dentostomella translucida* is a nematode parasitizing the digestive system of great gerbils [60, 61]. Kamranrashani et al. [62] reported that great gerbils are host for several species of cestodes and nematodes in Golestan province. Furthermore, dwarf tapeworm *Hymenolepis nana*, which is a cyclophyllidean zoonotic enteric parasite, known to be occurred in different rodents, including gerbils, of Golestan and Razavi Khorasan provinces in north of Iran [63].

Contact zones create candidate habitats where future researches could examine the extent of ecological divergence between the known lineages [64, 65]. Therefore, increasing knowledge regarding the rodent distribution and effective environmental variables, the patterns of rodent populations, colonization and movements, and finally, the preparation of risk maps for making decisions against zoonotic diseases and development are of great concern.

## Conclusion

The present study provided projections of the potential geographic distribution of the two subspecies of great gerbil, with a fundamental role in the epidemiology of zoonoses. For *R*. *o*. *sargadensis* distributed across most areas of Iran, southern Afghanistan and western Pakistan, the sampling area represents more than half of the geographic range of the subspecies, and the resulting model can potentially be of relatively good quality. However, this is not the case for *R*. *o*. *sodalis*. This subspecies is distributed across northeastern Iran, Turkmenistan and northern Afghanistan. Cumulatively, future studies should consider covering the whole geographical distribution range, especially identifying contact zones, population structure, and comprehensive distribution samplings for genetic studies are required to clarify the taxonomic status of this species. Using a more comprehensive dataset for ecological niche modeling and habitat evaluation will increase the precision of the models and estimate the probable future distribution of the species considering the role of climate changes affecting the environmental variables.

## Supporting information

S1 Appendix Point localities for *Rhombomys opimus* subspecies in Iran. (XLSX)

## References

[1] Abai MR, Oshaghi MA, Tajedin L, Rassi Y, Akhavan AA. Geographical distribution and ecological features of the great gerbil subspecies in the main zoonotic cutaneous leishmaniasis foci in Iran. Asian Pac J Trop Med. 2010; 3(10):800–3. doi: 10.1016/S1995-7645(10)60192-7

[2] Musser GG, Carleton MD. Superfamily Muroidea. In: Wilson DE, Reeder DM, editors. Mammal Species of the World: a taxonomic and geographic reference, 3rd Edition, John Hopkins University Press; 2005: pp. 894–1531.

[3] Denys C. Rhombomys opimus. In: Wilson DE, Lacher TE Jr, Mittermerier RA, editors. Handbook of Mammals of the World. Vol. 7. Rodents II, Lynx Edicion, Spain, Barcelona, 2017. pp. 649–50.

[4] Nowak R. Walker’s mammals of the world, 6th edition. Baltimore, MD: Johns Hopkins University Press; 1999.

[5] Gao M, Li Q, Cao C, Wang J. Spatial distribution and ecological environment analysis of great gerbil in Xinjiang Plague epidemic foci based on remote sensing. In: IOP Conference Series: Earth and Environmental Science. IOP Publishing; 2014: 17(1): p. 012265.

[6] Rogovin K, Randall JA, Kolosova I, Moshkin M. Social correlates of stress in adult males of the great gerbil, *Rhombomys opimus*, in years of high and low population densities. Horm Behav. 2003; 43(1):132–9. doi: 10.1016/s0018-506x(02)00028-4 12614643

[7] Ziaei H. A field guide to the mammals of Iran. Tehran, Iran: Iran. Wildlife Center Press; 2009.

[8] Oshaghi MA, Rassi Y, Tajedin L, Abai MR, Akhavan AA, Enayati A, et al. Mitochondrial DNA diversity in the populations of great gerbils, *Rhombomys opimus*, the main reservoir of cutaneous leishmaniasis. Acta Trop. 2011; 119(2–3):165–71. doi: 10.1016/j.actatropica.2011.05.010 21683054

[9] Ellerman JR. The families and genera of living rodents. Vol II- Muridae. 1941.

[10] Bakhshi H, Oshaghi MA, Abai MR, Rassi Y, Akhavan AA, Mohebali M, et al. MtDNA cytb structure of *Rhombomys opimus* (Rodentia: Gerbellidae), the main reservoir of cutaneous leishmaniasis in the borderline of Iran-Turkmenistan. J Arthropod Borne Dis. 2013; 7(2):173–84. 24409443 PMC3875884

[11] Corbet GB. The Mammals of the Palaearctic Region: A Taxonomic Review. British Museum (Natural History), London, 314 pp. 1978.

[12] Reithinger R, Dujardin JC, Louzir H, Pirmez C, Alexander B, Brooker S. Cutaneous leishmaniasis. Lancet Infect Dis. 2007; 7(9):581–96. doi: 10.1016/S1473-3099(07)70209-8 17714672

[13] Wilschut LI, Addink EA, Heesterbeek JA, Dubyanskiy VM, Davis SA, Laudisoit A, et al. Mapping the distribution of the main host for plague in a complex landscape in Kazakhstan: An object-based approach using SPOT-5 XS, Landsat 7 ETM+, SRTM and multiple Random Forests. Int J Appl Earth Obs Geoinf. 2013; 23:81–94. doi: 10.1016/j.jag.2012.11.007 24817838 PMC4010295

[14] Hamidi K, Bueno-Marí R. Host-ectoparasite associations; the role of host traits, season and habitat on parasitism interactions of the rodents of northeastern Iran. J Asia Pac Entomol. 2021; 24(1):308–19. doi: 10.21203/rs.3.rs-42804/v1

[15] Mohebali M, Javadian E, Yaghoobi Ershadi MR, Akhavan AA, Hajjaran H, Abaei MR. Characterization of *Leishmania* infection in rodents from endemic areas of the Islamic Republic of Iran. EMHJ 2004; 10(4–5):591–599. 16335651

[16] Akhavan AA, Yaghoobi-Ershadi MR, Khamesipour A, Mirhendi H, Alimohammadian MH, Rassi Y, et al. Dynamics of Leishmania infection rates in *Rhombomys opimus* (Rodentia: Gerbillinae) population of an endemic focus of zoonotic cutaneous leishmaniasis in Iran. Bull Soc Pathol Exot. 2010; 103(2):84–9. doi: 10.1007/s13149-010-0044-1 20390397

[17] Oshaghi MA, Rasolian M, Shirzadi MR, Mohtarami F, Doosti S. First report on isolation of *Leishmania tropica* from sandflies of a classical urban Cutaneous leishmaniasis focus in southern Iran. Exp Parasitol. 2010; 126(4):445–50. doi: 10.1016/j.exppara.2010.05.020 20570590

[18] Parvizi P, Baghban N, Novin EA, Absavaran A. Detection, identification and molecular typing of *Leishmania major* in *Phlebotomus papatasi* from a focus of zoonotic cutaneous leishmaniasis in central of Iran. Exp Parasitol. 2010; 124(2):232–7. doi: 10.1016/j.exppara.2009.10.004 19854172

[19] Rassi Y, Oshaghi MA, Azani SM, Abaie MR, Rafizadeh S, Mohebai M, et al. Molecular detection of Leishmania infection due to *Leishmania major* and *Leishmania turanica* in the vectors and reservoir host in Iran. Vector Borne Zoonotic Dis. 2011; 11(2):145–50. doi: 10.1089/vbz.2009.0167 20575646

[20] Hamidi K, Nassirkhani M. Annotated checklist of fleas (Insecta: Siphonaptera) and lice (Insecta: Anoplura) associated with rodents in Iran, with new reports of fleas and lice. J Vector Borne Dis. 2019; 56(2):134–45. doi: 10.4103/0972-9062.263715 31397389

[21] Graham MH. Confronting multicollinearity in ecological multiple regression. Ecology. 2003; 84(11):2809–15. doi: 10.1890/02-3114

[22] Karami M, Hutterer R, Benda P, Siahsarvie R., Kryštufek B. Annotated check-list of the mammals of Iran. Lynx, new series. Prague: National Museum, 2008: 39(1): 63–102.

[23] Karami M, Ghadirian T., Faizolahi K. The atlas of the mammals of Iran. Iran Department of the Environment, Tehran, Iran; 2016: 292 pp.

[24] Kabir M, Hameed S, Ali H, Bosso L, Din JU, Bischof R, et al. Habitat suitability and movement corridors of grey wolf (Canis lupus) in Northern 495 Pakistan. PLoS One. 2017; 12(11):e0187027. doi: 10.1371/journal.pone.0187027 29121089 PMC5679527

[25] Mohammadi S, Ebrahimi E, Moghadam MS, Bosso L. Modelling current and future potential distributions of two desert jerboas under climate change in Iran. Ecol Inform. 2019; 52:7–13. doi: 10.1016/j.ecoinf.2019.04.003

[26] Phillips SJ, Anderson RP, Schapire RE. Maximum entropy modeling of species geographic distributions. Ecol Modell. 2006; 190(3–4):231–59. doi: 10.1016/j.ecolmodel.2005.03.026

[27] Elith J, H. Graham C, P. Anderson R, Dudík M, Ferrier S, Guisan A, et al. Novel methods improve prediction of species’ distributions from occurrence data. Ecography. 2006; 29(2):129–51. doi: 10.1111/j.2006.0906-7590.04596.x

[28] Phillips SJ, Dudík M. Modeling of species distributions with Maxent: new extensions and a comprehensive evaluation. Ecography. 2008; 31(2):161–75. doi: 10.1111/j.0906-7590.2008.5203.x

[29] Duan RY, Kong XQ, Huang MY, Fan WY, Wang ZG. The predictive performance and stability of six species distribution models. PloS One. 2014; 9(11):e112764. doi: 10.1371/journal.pone.0112764 25383906 PMC4226630

[30] Elith J, Phillips SJ, Hastie T, Dudík M, Chee YE, Yates CJ. A statistical explanation of MaxEnt for ecologists. Divers Distrib. 2011; 17(1):43–57. doi: 10.1111/j.1472-4642.2010.00725.x

[31] Elith J. Quantitative methods for modeling species habitat: comparative performance and an application to Australian plants. In: Quantitative methods for conservation biology. New York, Springer; 2000: pp. 39–58.

[32] Franklin J. Mapping species distributions: spatial inference and prediction. Cambridge University Press; 2010: pp. 21–32.

[33] Hamidi K, Mohammadi S, Eskandarzadeh N. How will climate change affect the temporal and spatial distributions of a reservoir host, the Indian gerbil (*Tatera indica*), and the spread of zoonotic diseases that it carries?. Evol Ecol Res. 2018; 19(2):215–26.

[34] Ghassemi-Khademi T, Khosravi R, Sadeghi S, Ebrahimi M. Historical, current, and future climate niche of the red dwarf honey bee across its native range. J. Apic. Res. 2021; 1–13. doi: 10.1080/00218839.2021.1892370

[35] Warren DL, Glor RE, Turelli M. ENMTools: a toolbox for comparative studies of environmental niche models. Ecography. 2010; 33(3):607–11. doi: 10.1111/j.1600-0587.2009.06142.x

[36] Warren DL, Glor RE, Turelli M. Environmental niche equivalency versus conservatism: quantitative approaches to niche evolution. Evolution 2008; 62(11):2868–83. doi: 10.1111/j.1558-5646.2008.00482.x 18752605

[37] Schoener TW, Gorman GC. Some niche differences in three Lesser Antillean lizards of the genus *Anolis*. Ecology. 1968; 49(5):819–30. doi: 10.2307/1936533

[38] Gholamrezaei M, Mohebali M, Hanafi-Bojd AA, Sedaghat MM, Shirzadi MR. Ecological niche modeling of main reservoir hosts of zoonotic cutaneous leishmaniasis in Iran. Acta Trop. 2016; 160:44–52. doi: 10.1016/j.actatropica.2016.04.014 27150212

[39] Mohammadi Z, Darvish J, Haddad F, Ghorbani F. A karyological study of some murid rodents (Rodentia: Muridae) of Iran. PBS. 2012; 2(1):30–39.

[40] Varasteh-Moradi H. Study on intra-species variation of great gerbils (Rhombomys opimus) in Kalaleh, Turkmen-Sahara Iran. M.Sc. Thesis. Tarbiat Modares University, Tehran, Iran. 1998.

[41] Akhavan AA, Yaghoobi-Ershadi MR, Shirani-Bidabadi L. Karyosystematic and morphometric characterization of Rhombomys opimus and Meriones libycus the main reservoirs of zoonotic cataneous leishmaniasis in the endemic foci of Isfahan province, Iran. In: Proceedings of the 12th Congress of Infective and Tropical Diseases, Tehran; 2004: p. 22.

[42] Pardini R, de Souza SM, Braga-Neto R, Metzger JP. The role of forest structure, fragment size and corridors in maintaining small mammal abundance and diversity in an Atlantic forest landscape. Biol Conserv. 2005; 124(2):253–66. doi: 10.1016/j.biocon.2005.01.033

[43] Hamidi K, Hamidi A, Bueno-Marí R. Potential infections and zoonotic diseases transmitted by small laboratory mammals. In: Duncan LT, editor. Advances in Health and Disease. Volume 36, chapter 3; 2021. pp. 111–42. ISBN: 978-1-53619-569-9

[44] Wheeler DC, Waller LA. Mountains, valleys, and rivers: the transmission of raccoon rabies over a heterogeneous landscape. J Agric Biol Environ Stat 2008; 13(4):388–406. doi: 10.1198/108571108x383483 20396631 PMC2854036

[45] Lizhi Z, Yong M, Diqiang L. Distribution of great gerbil (*Rhombomys opimus*) in China. Dong Wu Xue Bao 2000; 46(2):130–7.

[46] Randall JA, Rogovin K, Parker PG, Eimes JA. Flexible social structure of a desert rodent, *Rhombomys opimus*: philopatry, kinship, and ecological constraints. Behav Ecol. 2005; 16(6):961–73. doi: 10.1093/beheco/ari078

[47] Randall JA. Behavioural adaptations of desert rodents (Heteromyidae). Anim Behav. 1993; 45(2):263–87. doi: 10.1006/anbe.1993.1032

[48] Randall JA. Convergences and divergences in communication and social-organization of desert rodents. Aust J Zool. 1994; 42(4):405–33.

[49] Shenbrot GI, Krasnov BR, Rogovin K. Spatial ecology of desert rodent communities. Berlin: Springer; 1999.

[50] Ari TB, Neerinckx S, Gage KL, Kreppel K, Laudisoit A, Leirs H, et al. Plague and climate: scales matter. PLoS Pathog. 2011; 7(9):e1002160. doi: 10.1371/journal.ppat.1002160 21949648 PMC3174245

[51] Rassi Y, Sofizadeh A, Abai MR, Oshaghi MA, Rafizadeh S, Mohebail M, et al. Molecular detection of Leishmania major in the vectors and reservoir hosts of cutaneous leishmaniasis in Kalaleh District, Golestan Province, Iran. J Arthropod Borne Dis. 2008; 2(2):21–7.

[52] Parvizi P, Ready PD. Nested PCRs and sequencing of nuclear ITS‐rDNA fragments detect three *Leishmania* species of gerbils in sandflies from Iranian foci of zoonotic cutaneous leishmaniasis. Trop Med Int Health. 2008; 13(9):1159–71. doi: 10.1111/j.1365-3156.2008.02121.x 18631311

[53] Naumov NP, Lobachev VS. Ecology of desert rodents of the USSR (jerboas and gerbils). In: Parkash I, Ghosh CK, editors. Rodents in desert environments. The Hague: Junk; 1975: 465–598.

[54] Rapoport LP, Melnichuck EA, Orlova LM, Nuriev KK. Comparative analysis of the flea fauna and its epizootic importance in the deserts of Southern Kazakhstan. Entomol Rev. 2010; 90(8):1003–13.

[55] Tajedin L, Rassi Y, Oshaghi MA, Telmadarraiy Z, Akhavan AA, Abai MR, et al. Study on ectoparasites of *Rhombomys opimus*, the main reservoir of zoonotic cutaneous Leishmaniasis in endemic foci in Iran. Iran J Arthropod Borne Dis 2009; 3(1):41–5. 22808371 PMC3385522

[56] Macdonald D. The Encyclopedia of Mammals, Facts on File Publications. New York; 1984.

[57] Hanafi-Bojd AA, Shahi M, Baghaii M, Shayeghi M, Razmand N, Pakari A. A study on rodent ectoparasites in Bandar Abbas: the main economic southern seaport of Iran. J Environ Health Sci Eng. 2007; 4(3):173–6.

[58] Telmadarraiy Z, Vatandoost H, Mohammadi S, Akhavan AA, Abai MR, Rafinejad J, et al. Determination of rodent ectoparasite fauna in Sarpole-Zahab district, Kermanshah Province, Iran, 2004–2005. J Arthropod Borne Dis. 2007; 1(1):58–62.

[59] Pakdad K, Ahmadi NA, Aminalroaya R, Piazak N. A study on rodent ectoparasites in the North district of Tehran, Iran during 2007–2009. J Paramed Sci. 2012; 3(1):42–6. doi: 10.22037/jps.v3i1.2923

[60] Schulz RE, Landa DM. Parasitic worms of the great gerbil *Rhombomys opimus* Licht (in Russian). Vest Microbiol Epidem Parazit. 1934; 13:305–15.

[61] Shleikher EI, Samsonova AV. The helminth fauna of Rhombomys opimus in Uzbekistan. Petrov AM, editor. Papers on helminthology presented to academician Skryabin KI on his 75th birthday. Akademiya Nauk SSSR, Moscow; 1953: pp. 770–3.

[62] Kamranrashani B, Kia EB, Mobedi I, Mohebali M, Zarei Z, Mowlavi G, et al. Helminth parasites of *Rhombomys opimus* from Golestan Province, northeast Iran. Iranian J Parasitol. 2013; 8(1):78–84. 23682264 PMC3655244

[63] Mirjalali H, Kia EB, Kamranrashani B, Hajjaran H, Sharifdini M. Molecular analysis of isolates of the cestode *Rodentolepis nana* from the great gerbil, *Rhombomys opimus*. J Helminthol. 2016; 90(2):252–5. doi: 10.1017/S0022149X15000115 25779770

[64] Rissler LJ, Smith WH. Mapping amphibian contact zones and phylogeographical break hotspots across the United States. Mol Ecol. 2010; 19(24):5404–16. doi: 10.1111/j.1365-294X.2010.04879.x 21054603

[65] Ghaedi Z, Badri S, Vaissi RS, Javidkar M, Ahmadzadeh F. The Zagros Mountains acting as a natural barrier to gene flow in the Middle East: more evidence from the evolutionary history of spiny-tailed lizards (Uromasticinae: Saara). Zool J Linn Soc. 2020: zlaa113, doi: 10.1093/zoolinnean/zlaa113

